# The Effects of Distance, Time, and Nonspatial Factors on Hemodialysis Access in Qatar

**DOI:** 10.7759/cureus.58569

**Published:** 2024-04-18

**Authors:** Anas Al Halabi, Abdullah Hamad, Hafedh Ghazouani, Mohamad Alkadi, Elmukhtar Habas, Rania Ibrahim, Hassan Al-Malki, Abdul-Badi Abou-Samra

**Affiliations:** 1 Quality and Patient Safety, Hamad Medical Corporation, Doha, QAT; 2 Nephrology, Hamad Medical Corporation, Doha, QAT; 3 Nephrology, Hamad General Hospital, Doha, QAT; 4 Internal Medicine, Hamad General Hospital, Doha, QAT; 5 Medicine, Qatar Metabolic Institute, Hamad Medical Corporation, Doha, QAT

**Keywords:** spatial factors, travel distance and time, qatar hemodialysis, hemodialysis center, dialysis

## Abstract

Background

A long distance and time spent traveling to a hemodialysis (HD) center and other factors, such as comorbidities, can significantly impact HD patient compliance, satisfaction, and cost. Uncertainty about HD-dependent patients’ geographical location may lead to inappropriate distribution of HD centers. The present study investigates travel time, distance, and nonspatial factors affecting HD center accessibility within a 30-km radius in the State of Qatar.

Materials and methods

The study included all HD-dependent patients residing in Qatar between March 1, 2020, and December 31, 2021. There were 921 patients dialyzed in six HD centers across Qatar. Our methodology incorporated descriptive and analytical cross-sectional designs to accurately identify the shortest routes and quickest travel times. We used two applications (Maptive {Vancouver, WA: BatchGeo LLC} and Google Maps {Mountain View, CA: Google LLC}) and marked a driving distance of 30 km as the main assessment scale and measurement standard, allowing optimum spatial accessibility determination.

Results

On average, patients traveled approximately 19±4.2 km, requiring almost 17.6±3.4 minutes to reach the assigned HD center three times per week. Based on geographic-spatial accessibility analysis, patients living in Umm Salal drove 31.4±3.5 km in 32.4±4.7 minutes, Al Daayen patients drove 30.2 km in 25.3 minutes, and others even drove more than 70 km to access HD sessions. Approximately 37.8% of Qatar’s municipalities had no HD centers within their boundaries, but nearly 47% of HD-dependent patients lived in those municipalities. Additionally, some municipalities had HD centers; however, their general population density was less than 100 inhabitants/km^2^, and they had relatively few patients requiring regular HD. We noted a statistically significant correlation between the patients’ residences and the locations of HD centers, whether they were located within or outside municipalities. Also, nonspatial factors may have affected the likelihood of reaching a hemodialysis center within a 30-km distance, including two or more comorbid conditions, having HD for at least five years, living in a municipality with more than 1,000 inhabitants/km^2^, being female, and attending dialysis centers that are more than 30 km away.

Conclusion

Although the available HD centers were sufficient for the present number of patients requiring HD, HD center locations did not match the patients’ distribution, leading to difficulties for some patients. Understanding the impact of this geographic mismatch, population density, and other spatial factors helps significantly improve patient care and satisfaction at minimal cost. Furthermore, considering all these factors is crucial when planning new centers to achieve higher satisfaction and compliance as well as better health care.

## Introduction

End-stage renal disease (ESRD) patients require either kidney transplantation or maintenance dialysis to survive [1]. Hemodialysis (HD) is the primary maintenance therapy, which usually consists of three sessions per week in a stable HD-dependent patient [2]. HD is commonly available in specialized, well-equipped centers; however, home HD currently takes place in a number of different countries, including Qatar. Research indicates that easy access and convenient HD service locations can enhance HD-dependent patients’ overall experience and satisfaction, as well as improve outcomes [3].

In 2021, the population of Qatar was approximately 2,688,000 (with an annual growth rate of 1.73%), distributed over eight municipalities. Doha is the capital where more than 1.3 million people live, comprising 40% of the population. The other municipality's populations range from 107,530 to 1,075,294 people [4]. According to the Ministry of Urban Planning and Development’s Climate Change Strategy Assessment, 99.2% of Qatar’s population lives in urban areas [5]. Additionally, the country’s urbanization rate is currently 2.41% per year, suggesting the urbanization rate is changing [6].

Researchers have previously explored connections between the geographical distribution of health service centers and health care needs, giving considerable attention to the spatial or geographic aspects of service accessibility [7]. The availability of hospitals and dialysis facilities are crucial factors in identifying regions with limited healthcare access [8]. The reachability measure assesses travel difficulty, whereas the spatial accessibility measure evaluates healthcare accessibility and availability to reflect potential benefits [9]. Geography can offer valuable insights into the spatial dimensions of access to services, which positively impacts patient satisfaction and outcomes [10]. It is important to determine whether the distance to a health center providing the service affects healthcare accessibility [11]. Spatial analysis and geographic information systems are instruments for studying and determining the location of healthcare facilities [12].

Qatar has a low prevalence of dialysis-dependent patients because most of the population is young. In December 2021, Qatar had 1,184 dialysis-dependent patients. There were 921 (78%) HD-dependent patients, and 263 (22%) were peritoneal dialysis patients [13]. The ESRD patient count in Qatar is expected to increase annually by 5.67% on average, reaching an estimated 1,611 patients in 2030 [13]. The COVID-19 pandemic was a real challenge for available HD services in terms of accommodating an increased need for urgent HD in Qatar [14,15]. Hence, allocating sufficient resources to improve current HD centers and new HD centers is essential. Furthermore, considering the centers’ geographic distribution and functional characteristics is necessary before 2030 to ensure sufficient patient access to the required treatment [16].

Therefore, we aimed to investigate the travel distance and time required to access ambulatory HD centers and to identify nonspatial factors determining access to the available HD centers within a 30-km radius. Moreover, we hope to provide a vision for existing HD center distribution and create new HD service centers in Qatar based on population density and patient location to provide the most efficient clinical care at the lowest possible cost.

Ethical approval

The Office of the Chief Quality Officer of Hamad Medical Corporation (HMC) granted approval for this research as a quality improvement initiative (decision letter no. 020223). Hence, this study does not need approval from the Institutional Review Board (IRB). This research collected data on many demographic factors of the patients, including their age, home address, gender, name and location of the hemodialysis facility they attended, their overall satisfaction with the service and distance, and the time it took for them to travel to and from the HD center. All patients have completed and signed the permission form, authorizing the release of the necessary information, and the results can be published anonymously.

## Materials and methods

We collected the data from Qatar’s official population data source (www.data.gov.qa), extracting valuable insights from the electronic medical records of 921 HD-dependent patients who received dialysis in six outpatient ambulatory HD centers. The HD centers were as follows: two centers in Al Wakrah, one in Doha, one in Al Shahaniya, one in Al Shamal, and one in Al Khor. The collected data included age, comorbidities, living in the municipality, gender, dialysis unit structure, number of dialysis stations, location, map direction, population density, and municipality of the HD center. We also gathered all patients’ home and HD center addresses.

Euclidean distance measures the shortest way or path between two locations in a smooth n-dimensional space. We used Euclidean distance to make precise planar calculations, integrating geometric and mathematical techniques. After collecting the included patients’ addresses and HD service locations, we calculated the shortest distances and fastest travel times. After that, we converted distance and duration into Euclidean distance measures using the Google Maps API (Mountain View, CA: Google LLC) and Maptive applications (Vancouver, WA: BatchGeo LLC).

Computing transportation vehicles’ average speed

**Figure 1:**
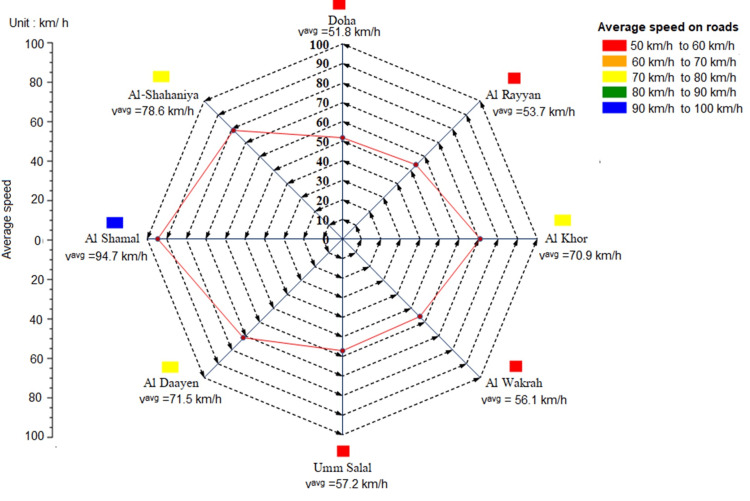
Estimating representative urban auto-driving based on dialysis patients’ data for each municipality’s weighted average speed. This figure is created by the authors of this study with the help of Microsoft software version 365.

In Qatar, patients use private vehicles, taxis, or ambulances to travel to HD treatment. Patients rarely used public transportation to get to their dialysis centers. We used road network mapping and geospatial data to estimate mobility and vehicle speed restrictions. To calculate the weighted average speed, we calculated the road speed based on the shortest time and distance for 30 patients in each municipality. Considering a range of factors, including traffic jams and time of day, we calculated the weighted minimum speed based on the GPS-based data. The weighted average vehicle speed was between 50 and 100 km/h. Figure 1 shows the representative estimate of urban traffic based on data from dialysis patients.

By examining the patient’s visit history, we were able to accurately determine their exact location using street coordinates. To determine the dialysis center location, we used the Google Maps API web service in conjunction with the Hemodialysis Center Guide. We then created a custom results map using the Maptive mapping application. We integrated Google Maps into mapping tools, ignored transportation modes, and focused solely on speed limits on city streets. After that, we calculated the shortest distance in kilometers and the time in minutes with a distance/time calculator based on Euclidean measurements, specifically using Google Maps as a reference. We organized the data by the municipality by applying the following weighted average formula:

bar(d) = d ̅ = Σ [(di×Pi)] / Σ [Pi] (1)

bar(t) = t ̅ = Σ [(ti×Pi)] / Σ [Pi] (2)

Here, d-bar (d ̅) was calculated as the weighted average of the distance between the dialysis center and the patient's residence in a unified municipal zone. In contrast, t-bar (t ̅) was calculated as the weighted average of the time between the dialysis center and the patient's residence. Variables (di) and (ti) represent the distance and time, respectively, between a dialysis center and a patient's place of residence at various data points within the population. The variable (Pi) is a unique identifier assigned to each patient in a municipality, with the number of data points in the population ranging from one to n. Additionally, patients' residences from dialysis centers were categorized into two distance zones, each with a maximum limit of 30 km.

Statistical analysis

We used Stata version 16.1 (College Station, TX: Stata Corp) for exploratory data analysis and descriptive statistics, using the mean, median, standard deviation, range, and percentile for continuous data as descriptive measures. We performed a binary logistic regression analysis and developed a predictive model to identify nonspatial factors contributing to the chances of reaching dialysis centers within a 30-km radius.

We included age, race, gender, locations of dialysis treatment, and the distance between the patient’s home and the hemodialysis center in the statistical analysis. As a dummy variable, we set a driving distance of less than 30 km. This binary variable takes a score of zero if the distance is less than 30 km and a score of one if the distance is more than 30 km. We then treated it as an independent variable, using Spearman’s correlation to find the relationship between two variables within three groups. We considered p≤0.05 to be statistically significant.

## Results

Among the participants, 60% were males, of whom 48% were of Qatari nationality. The mean age of the patients was 59.6±12.8 (SD). Approximately 70% were >55 years old, and 77% of patients had two or more comorbidities. Approximately 78% of the participants had been receiving HD for at least three years. Data analysis of age and comorbidity showed no significant differences across patients in different HD centers. Table 1 summarizes patient demographics and clinical characteristics.

**Table 1:** Demographic and clinical characteristics of 921 hemodialysis patients.

Category subcategory patients	Number of patients (%)
Age	
0-49 years	239 (26%)
50-64 years	332 (36%)
>65 years	350 (38%)
The average age±SD	59.6±12.8 years
Comorbidities	
0-1 comorbidity	212 (23%)
>2 comorbidities	709 (77%)
Nationality	
Qatari	442 (48%)
Non-Qatari	479 (52%)
Gender	
Male	553 (60%)
Female	368 (40%)
Hemodialysis treatment time	
0-2 years	203 (22%)
3-5 years	276 (30%)
>5 years	442 (48%)
The average duration of hemodialysis±SD	3.1±1.9 years

There were six ambulatory HD centers across five municipalities in Qatar. The biggest HD center was the Fahad Bin Jassim Kidney Center (FBJKC) in Doha, where 52% of HD-dependent patients received dialysis at 92 stations daily. The remaining 48% of patients received HD at the five other centers. These centers’ capacity ranged between 10 and 23 HD stations. The FBJKC HD center had three daily shifts for the whole week except Friday whereas other HD centers had only one or two shifts. This difference between the number of shifts was due to the number of patients and available staff in each HD center.

The total number of available HD stations in 2021 was 178, serving a maximum of 544 HD sessions when each station was used for three sessions every 24 h. This means that 3264 HD sessions could be conducted weekly if the available HD stations were fully utilized. Only four of the seven HD centers had the workforce to operate three daily dialysis shifts. Hence, instead of conducting 3264 HD weekly sessions, they conducted 2934 sessions for 978 ESRD patients/week, representing 86% of the total capacity (Table 2).

**Table 2:** Number of dialysis stations and shifts in outpatient ambulatory dialysis centers in 2021. HMC: Hamad Medical Corporation; FBJKC: Fahad Bin Jassim Kidney Center

Dialysis unit/HMC dialysis facilities capacity	Volume contribution %	No. shifts per day	No. of stations	Currently occupied unit capacity %
FBJKC	52%	3	92	90%
Al Khor Hospital	13%	2	23	67%
Al Wakra Hospital	10%	3	18	100%
Al Wakra Health Center	13%	3	23	100%
Al Sheehaniya Health Center	7%	2	12	67%
Al Shamal Medical Center	6%	2	10	67%
Total or average	100%	3	178	86%

Geographical accessibility

The state of Qatar consists of eight municipalities. Among these municipalities, Al Rayyan had the highest proportion of dialysis patients at 34%. The second-highest number of patients, accounting for 31% of the total, was in the municipality of Doha, which had the highest population density with over 1,000 inhabitants/km. The remaining six municipalities accounted for 35% of dialysis patients. Notably, more than half (52.3%) of HD-dependent patients in Qatar lived in municipalities without an HD center. Additionally, more than 63% of HD patients in Qatar received dialysis in centers outside their local municipalities. Table 3 shows the location of HD distribution of population density and municipality.

**Table 3:** Placement of dialysis centers is based on population density and municipality. *The 2021 census reveals Qatar's population as 2,688,235. **The number of hemodialysis patients in Qatar in 2021 was 921.

Municipality	Percentage of population distribution by municipality*	Dialysis patients living in the municipality (%)**	Population density (inhabitants per km^2^)	Percentage of patients receiving treatment outside their municipality of residence
Doha*	40%	286 (31%)	≥1000	34%
Al Rayyan	25%	313 (34%)	100 ≤ density <1000	100%
Al Khor*	8%	38 (4%)	<100	7%
Al Wakrah*	12%	140 (15%)	100 ≤ density <1000	3%
Um Salal	4%	48 (5%)	100 ≤ density <1000	100%
Al Daayen	2%	78 (8%)	100 ≤ density <1000	100%
Al Shamal*	0.4%	7 (0.7%)	<100	0%
Al Shahaniya*	8%	11 (1%)	<100	100%

Overall, patients requiring HD traveled an average of 19.1±4.2 km to access an HD center, taking an average of 17.6±3.4 minutes. More than half of the HD patients (62%) had a travel distance of less than 30 km. At the 90th percentile of HD patients, the distance traveled was <40.3 km and only 7.1% traveled 87.1 km.

**Figure 2:**
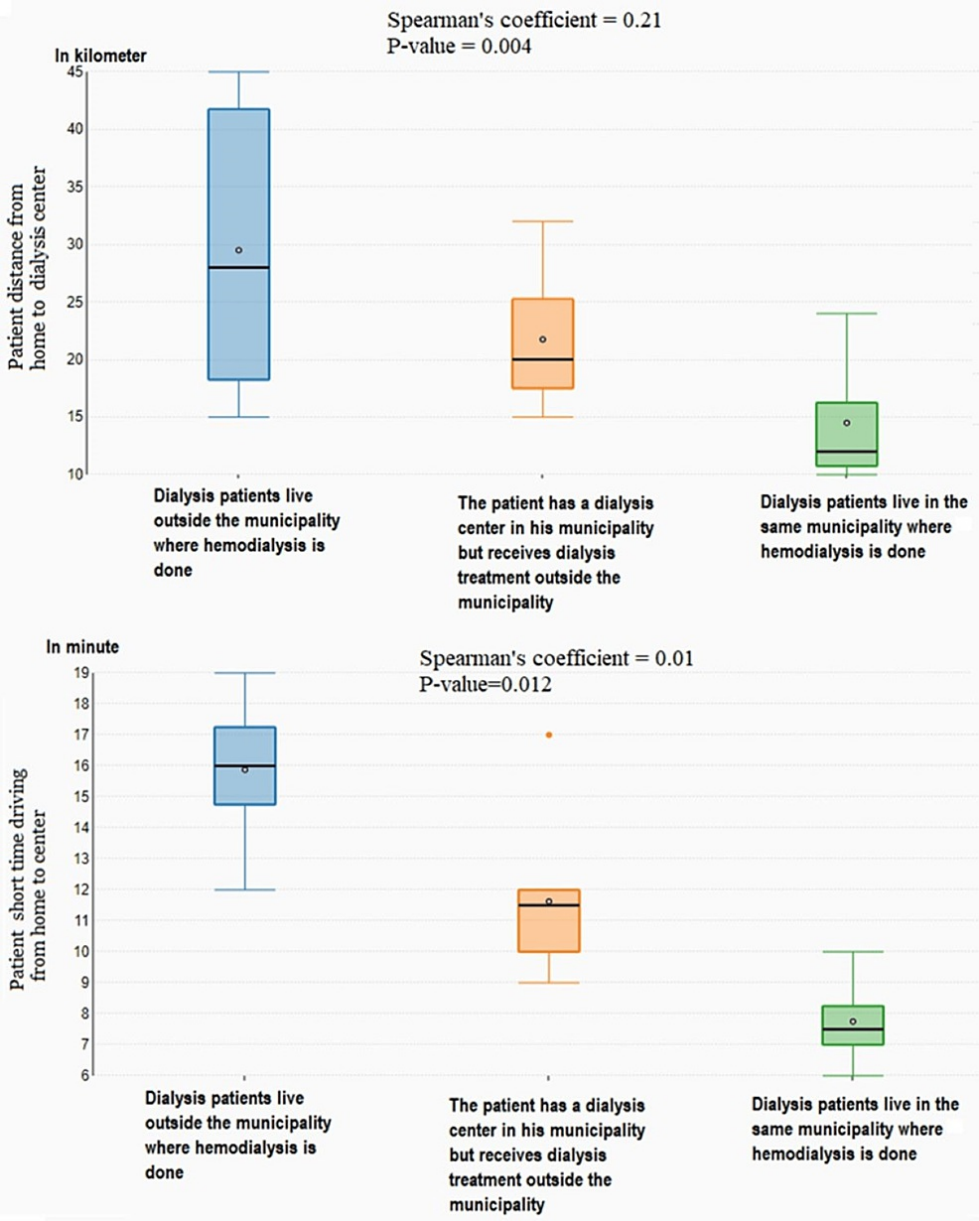
Map of various dialysis centers in an interactive format. This figure is created by the authors of this study with the help of Microsoft software version 365.

Patients living in municipalities without dialysis centers traveled an average of 28 km with an average travel time of 23.2 minutes. Conversely, patients who lived in municipalities that had HD centers traveled an average of 14.5 km with an average travel time of 15 minutes. We found a statistically significant positive relationship between HD center locations, distance traveled (Spearman coefficient=0.21, p=0.004), and time (Spearman coefficient=0.01, p=0.012). Figure 2 shows the distance and time required to reach the assigned HD center.

Analysis of spatial disparities

Analysis of spatial disparities revealed that the average distance traveled by HD patients living in Doha was 15.1±2.5 km with a range of 8.6-61.5 km and an average travel time of 17.4±2.5 minutes with a range of 5.2-27.5 minutes. About two-thirds of HD patients who lived in Doha traveled less than 30 km. In Al Rayyan municipality, HD patients traveled an average of 19.6±5.3 km, ranging from 7.4 to 81.7 km, with an average travel time of 21.8±3.1 minutes, ranging from 4.1 to 39.5 minutes. However, 83% of HD patients in Al Rayyan traveled fewer than 30 km. HD patients living in Al Khor traveled an average distance of 9.7±0.8 km, ranging from 5.2 to 89.7 km, with an average travel time of 8.2±1.1 minutes, ranging from 3.0 to 27.5 minutes. HD patients living in Al Wakra travel a distance of 22.4±4.1 km, ranging from 5.6 to 68.5 km, with an average travel time of 23.9±3.4 minutes, ranging from 3.5 to 46.6 minutes. Notably, 71% of HD patients living in Al Wakra traveled fewer than 30 km. HD patients living in Um Salal traveled an average distance of 31.4±3.5 km, ranging from 5.2 to 89.7 km, and an average travel time of 32.9±4.7 minutes, ranging from 4.1 to 44.7 minutes. Half of these patients traveled more than 30 km each HD day. Patients living with HD in Al Dayyan traveled an average distance of 30.2±2.8 km, with a range of 7.8-92.3 km, and an average time of 25.3±3.6 minutes, with a range of 5.0-31.3 minutes. Approximately two-thirds of these patients traveled more than 30 km. HD patients living in Al Shamal traveled an average distance of 7.5±0.9 km, with a range of 4.2-23.6 km and an average time of 6.1±0.6 minutes, with a range of 3.1-19.5 minutes. None of these patients traveled more than 20 km. Finally, HD patients in Al Sheehaniya traveled an average distance of 27.1±3.5 km, with a range of 9.1-91.2 km, and an average time of 20.6±2.9 minutes, with a range of 4.4-40.2 minutes. Unfortunately, 77% of these patients traveled more than 30 km each dialysis day. Table 4 summarizes these travel distance and time data.

**Table 4:** Travel distance (km) and time (minutes) for 921 patients to dialysis center. *Municipality with a dialysis facility. **Data reflects mean±SD (standard deviation). The 2021 census reveals Qatar's population as 2,688,235.

Municipality	%	Average travel times by taking the fastest route (minutes)**	Distance traveled (km) to the dialysis center where patients receive treatment*						
			<10 km	10-19 km	20-29 km	30-39 km	40-49 km	>50 km	Average distance between dialysis facilities and patients' residences (km)**
Doha*	31%	17.4±2.5	46%	15%	7%	20%	4%	8%	15.1±2.5
Al Rayyan	34%	21.8±3.1	5%	61%	17%	10%	3%	4%	19.6±5.3
Al Khor*	4%	8.2±1.1	83%	5%	1%	4%	3%	4%	9.7±0.8
Al Wakra*	15%	23.9±3.4	21%	37%	13%	12%	11%	6%	22.4±4.1
Umm Salal	5%	32.9±4.7	8%	20%	20%	42%	7%	3%	31.4±3.5
Al Daayen	8%	25.3±3.6	4%	20%	9%	32%	20%	15%	30.2±2.8
Al Shamal*	0.7%	6.1±0.6	59%	41%	-	-	-	-	7.5±0.9
Al Sheehaniya*	1%	20.6±2.9	5%	7%	11%	28%	21%	28%	27.1±3.5
All patients	100%	17.6±3.4	38%	19%	5%	16%	11%	11%	19.1±4.2

**Figure 3:**
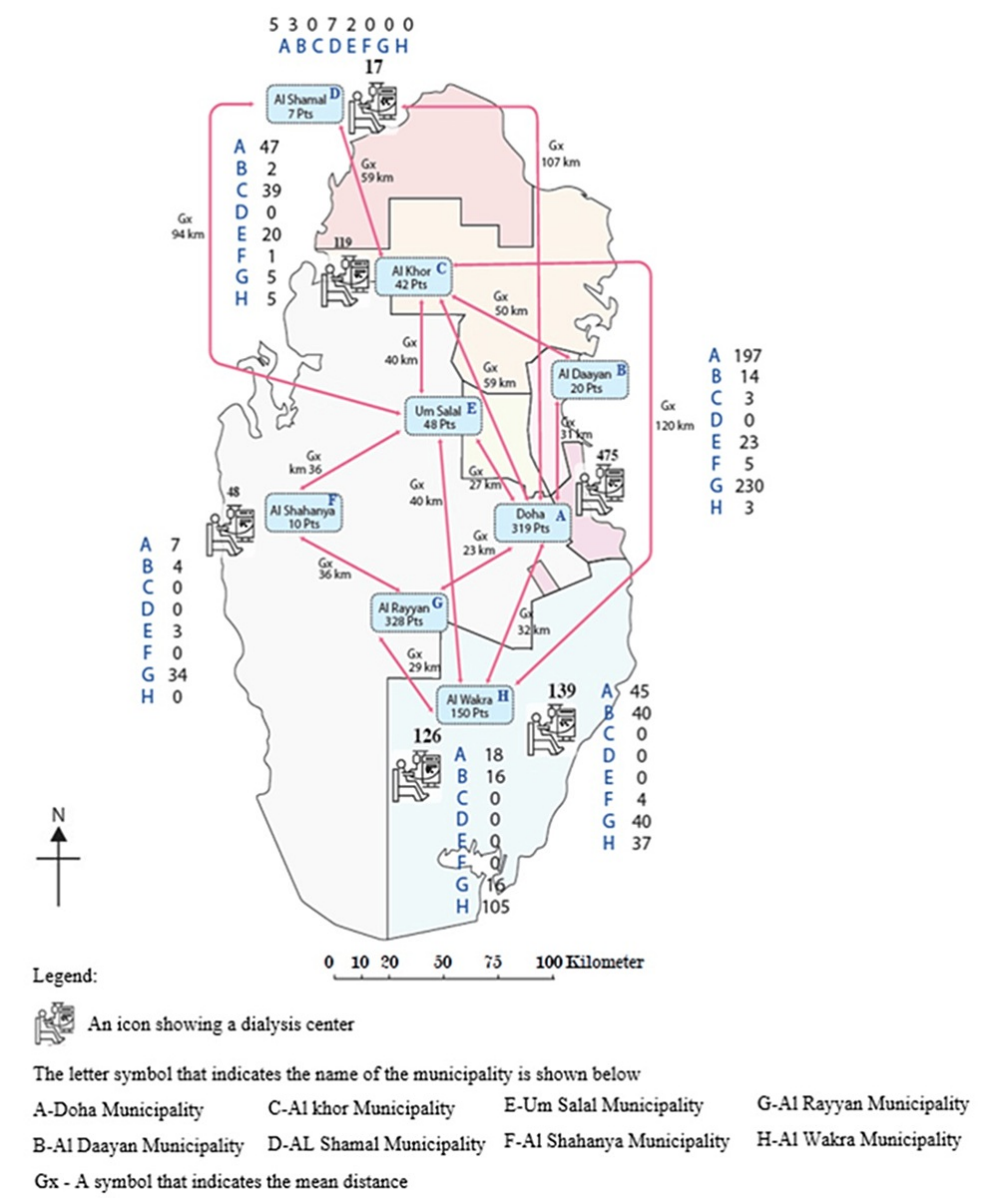
Map illustrating various dialysis centers in an interactive format. This figure is created by the authors of this study with the help of Microsoft software version 365.

Using the Déclic Geometric Software version 5.23.1.8 (INRA: Thonon-les-Bains, France) on a Windows platform revealed that HD centers formed a distinctive concave hexagonal shape with irregular curves and six sides with a precise midpoint, diameter, and beam coordinates that accurately located each center. The analysis indicated that the dialysis centers were 50.1 km from the hexagon’s center, which was located in the northwest area of Al Rayyan municipality. Moreover, we discovered that the primary diameter representing the longest distance between the two centers was 129.5 km whereas the minimum diameter representing the shortest distance between the two centers was 6.7 km. Figure 3 shows these distance variations on the map of Qatar.

Statistical results show that some spatial and nonspatial factors significantly influence access to HD centers within 30 km of homes. These factors include the presence of two or more comorbid conditions (95% confidence interval [CI]: 4.22-9.10, odd ratio [OR]=6.19, p=0.002), HD for at least five years (CI: 1.08-1.6, OR 1.14, p=0.034), living in an area with a population density of greater than 1,000 individuals per km^2^ (95% CI: 1.17-3.19, OR=1.58, p=0.001), being female (95% CI: 1.01-1.40; OR=1.08, p=0.008), having Qatari nationality (95% CI: 1.07-1.83, OR=1.4, p=0.017), and attending a dialysis center with at least 40 stations (95% CI: 1.15-2.34, OR=1.62, p=0.010).

Other factors can impact the likelihood of a patient’s inability to reach an HD center within 30 km. These factors include being under 50 years of age (95% CI: 0.31-0.66, OR=0.45, p=0.012), living in Umm Salal (95% CI: 0.28-0.87, OR=0.46, p=0.037), living outside the municipality where hemodialysis is performed (95% CI: 0.31-0.59, OR=0.43, p=0.001), and attending a dialysis center in the northwest part of the country (95% CI: 0.13-0.95, OR=0.71, p=0.001). Table 5 summarizes the results of our analysis.

**Table 5:** Displays odds ratios and confidence intervals from multiregression analysis of HD patient access to dialysis centers within 30 km. NS: not statistically significant; FBJKC: Fahad Bin Jassim Kidney Center

Factors considered	OR (CI)	p-Value
Age		
14-49 years	0.45 (0.31-0.66)	0.012
50-64 years	1.06 (0.78-1.45)	NS
>65 years	1	>65 years
Comorbidities		
0-1 comorbidity	1	0-1 comorbidity
Two or more comorbidity	6.19 (4.22-9.10)	0.002
Community affiliation		
Doha municipality	1	-
Doha municipality	1	-
Al Rayyan municipality	0.88 (0.44-1.29)	NS
Al Khor municipality	0.74 (0.37-1.46)	NS
Al Wakra municipality	0.78 (0.52-1.71)	NS
Umm Salal municipality	0.4 6 (0.24-0.87)	0.037
Al Daayen municipality	0.62 (037-1.05)	NS
Al Shamal municipality	0.39 (0.07-2.15)	NS
Al Sheehaniya municipality	1.55 (0.38-6.33)	NS
Nationality		
Non-Qatari	1	-
Qatari	1.4 (1.07-1.83)	0.017
Gender		
Male	1	-
Female	1.08 (1.01-1.4)	0.008
Dialysis unit structure		
In-center FBJKC	1	-
Al Wakra satellite	1.28 (0.88-1.86)	NS
Al Wakra Hospital	1.20 (0.87-1.72)	NS
Al Khor Hospital	1.12 (0.72-1.76)	NS
Al Sheehaniya satellite	1.08 (0.59-1.98)	NS
Al Shamal satellite	0.79 (0.31-2.04)	NS
The number of provided dialysis stations		
<10	1	-
In between 10 and 20 km	0.59 (0.15-1.90)	NS
In between 20 and 40 km	1.17 (098-1.62)	NS
>40 km	1.62 (1.15-2.34)	0.010
Based on localization and map-direction		
North	1.28 (0.48-1.36)	NS
West	0.71 (0.13-0.95)	0.000
East	1.27 (0.72-1.83)	NS
South	0.94 (0.66-1.25)	NS
The number of people per unit of area (population density)		
Density≤100	1	-
100	0.79 (0.38-2.91)	NS
Density≥1000	1.58 (1.17-3.19)	0.001
Place of residence		
Dialysis patients live in the same municipality where hemodialysis is done	1	-
Dialysis patients live outside the municipality where hemodialysis is done	0.43 (0.31-0.59)	0.0001
Hemodialysis treatment time		
0-2 years	1	-
3-5 years	0.92 (0.66-1.25)	NS
Above five years	1.14 (1.08-1.65)	0.034

## Discussion

Qatar’s Ministry of Public Health (MoPH) provides nephrology services and renal replacement therapies free of charge for Qatari and non-Qatari residents. There are only six public HD centers in Qatar fully covered financially by MoPH, and there is no private HD sector. Our study is the first to examine the accessibility of dialysis services in Qatar with a focus on travel distance and time. By mapping the availability of HD services in the eight municipalities, we identified areas with inadequate coverage and analyzed factors contributing to unequal distribution of health resources. Visual representation of our results is critical for effective dialysis resource planning and future government investments in dialysis.

We studied patient access to HD services using planar methods (Euclidean distance), focusing on geometric and mathematical approaches. As part of the evaluation, we assessed the physical accessibility (distance and travel time) of patients to the HD center [17]. During the analysis, we focused only on the value of the short drive distance. We used Maptive and Google Maps to determine the shortest distance and fastest achievable travel time. Furthermore, we examined the density of population distribution in Qatari municipalities, the number of ESRD-dependent HD patients, patients’ geographic locations, and transportation options. We included all these factors to ensure the results reflected all realistic conditions [18].

We recruited 921 ESRD-HD-dependent patients in six MoPH-owned outpatient dialysis centers in 2021 for the study, finding that these patients had to travel an average of 19.1±4.2 km to arrive at the assigned HD center, taking an average of 17.6±3.4 min. In this study, we opted for a target distance of 30 km to ensure that better-suited HD centers are available nationwide, and we thus used a driving distance of <30 km as a criterion for defining low spatial accessibility. According to the Foundation of International Standards Guidelines, the distance between a patient’s residence and their HD center should not exceed 40 km [19]. The average distance and time in this study were longer than those reported in the United States (16.4 km and 22 minutes) [20]. The minimum distance to access specialized hospital services in the United Kingdom ranges from 38.6 to 80 km [21]. Furthermore, value-based healthcare fundamentals guide dialysis delivery and other health services, warranting the best healthcare, patient safety, and satisfaction [22].

The study results indicated that the average distance traveled in the 90th percentile was 40.3 km and the maximum was 87.1 km, indicating that the location distribution of HD centers did not match HD patients’ locations. Recognizing this defect and correcting it will improve HD services. Maintaining a balance between the demand for HD services and patients’ numbers and locations will enhance patients’ quality of life by harmonizing the geographic location of a patient’s home and their HD center [23].

Data analysis shows that patients living within the dialysis centers’ location boundaries, like in Al Wakra municipality, had a clear advantage in accessing their HD center because of the shorter distance and arrival time. Patients hemodialyzed at HD centers in the same municipality as their residency traveled approximately 33% less than those dialyzed at HD centers located outside their residency municipality. Travel outside the municipality burdened the patient with extra travel distance by 51% and extra time. Patient choice was one major cause of these longer travel distances, for example, some patients insisted on being dialyzed in specific centers such as FBJKC, even if they had another HD center nearby. An Australian study showed that HD patients in major municipalities spent 90 minutes per week traveling in addition to the time spent receiving dialysis treatment. It also showed a higher travel burden for patients in their first year of treatment. The same study reported that factors such as the level of clinical care required during treatment, availability and structure of hemodialysis services, dialysis capacity constraints and staffing, patient preferences, and mode of transport may contribute to longer travel times. Our search did not find any reported data indicating that factors such as age, gender, or education affect patients' compliance with hemodialysis treatment [24].

This study revealed significant disparities in dialysis service accessibility across the eight municipalities, indicating that accessibility to dialysis services varied significantly. Because of their geographical locations, Umm Salal and Al Daayen HD patients needed help reaching dialysis centers. Specifically, patients who resided in Umm Salal traveled an average distance of 31.4 km with an average travel time of 32.9 minutes, whereas patients living in Al Daayen traveled 30.2 km, with an average time of 25.3 minutes. Therefore, due to the unavailability of HD centers in these municipalities, patients had to travel an average of 133% more than those in municipalities that had HD centers. Location and population density are among the factors that influence the proximity of HD centers in this study, as demonstrated by similar research from the United States [25].

Consistent with previous research by Velázquez et al., a sizable proportion (40%) of counties in the United States experienced a shortage of dialysis services from 2012 through 201,925. On average, patients had to travel 14.3 miles (22.8 km) to access dialysis services. According to a study conducted by Stephens et al., rural patients had to travel four times the distance traveled by urban patients to reach a dialysis facility (46 vs. 10.9 km) [26]. In Qatar, there were no dialysis centers in three of eight municipalities, representing 37.8% of the state’s total area per municipal distribution, which might affect the time and distance needed for patients to access HD.

The present study showed HD center distribution in a concave hexagonal shape with prominent and irregular curves. In addition, we found that these centers were located at the heart of the hexagon. The number of patients in Qatar requiring long-term dialysis for renal failure will reportedly increase by 5.67% annually [13]. Therefore, expanding or establishing new dialysis centers in specific locations depending on geographical and population distribution is crucial. The need for improved access to dialysis treatment in the Al Rayyan region is becoming increasingly urgent because the current capacity of nearby HD centers is currently overwhelmed. In response, the Health Authority has announced a plan to establish new HD centers in the Muaither and Al-Waab areas [27]. These initiatives should significantly improve the HD centers’ ability to deliver high-quality care to HD patients because 34% of the patients who need HD live in Al Rayyan. The results of a previous study by Richard et al. support this initiative [28].

Our study results show that areas with low population density (fewer than 100 inhabitants per km^2^), had better spatial accessibility than regions with a high population density (100-1000 or >1000 inhabitants per km^2^). A study by Ahmadi et al. shows a negative correlation between proximity to HD centers, the prevalence of HD, and the number of people [29].

The data analysis of the present study was significantly affected by factors such as living within 30 km of the HD center, which significantly affected HD service and patient quality of life. Multiregression analysis showed that other factors, such as having more than two comorbidities, having HD for more than five years, living in a municipality with more than one thousand people per square kilometer, and being female, significantly affected patients’ HD access and quality of life. Considering these factors could make assessing the accessibility of HD centers significantly more precise. The results of the present study are consistent with previous reports [30].

Limitations

This study has limitations that should be considered in future investigations. First, the spatial planning system took traffic into account, affecting travel distance and time, whereas Google Maps usually looks at fast pathways. Second, our sample size was small, and patients with extreme outlier values in travel distance were excluded, which may have affected the calculations. Third, some Qatari patients wanted to be dialyzed at HD centers far from their homes, which affected the estimated distance and time in this study. Fourth, future researchers should consider socioeconomic status and cultural barriers, which may also affect travel time and distance.

## Conclusions

Qatar had enough HD stations to meet demand in 2021 and possibly even beyond that, and compared to other countries, the majority of patients in Qatar live within a reasonable distance and travel time to their HD center. However, some patients still traveled far to receive HD. Patients had less access to HD services according to the statistics, primarily because of inadequate geographical placement of HD facilities in patients’ residential areas. However, patients’ desire to be dialyzed in a specific preferred HD facility that was located farther away and socioeconomic and cultural patterns, may have been a major cause. We recommend that Qatar’s health authorities consider patient geographical dispersion when building new HD facilities to provide adequate service. Furthermore, applying rules to assigning patients to HD centers would reduce travel time and distance, although it may cause some dissatisfaction.
